# Parkinson’s Disease Detection via Bilateral Gait Camera Sensor Fusion Using CMSA-Net and Implementation on Portable Device

**DOI:** 10.3390/s25123715

**Published:** 2025-06-13

**Authors:** Jinxuan Wang, Hua Huo, Wei Liu, Changwei Zhao, Shilu Kang, Lan Ma

**Affiliations:** School of Information Engineering, Henan University of Science and Technology, Luoyang 471000, China; wangjinxuan@stu.haust.edu.cn (J.W.); 9902846@haust.edu.cn (W.L.); zhchw@haust.edu.cn (C.Z.); 185300000021@stu.haust.edu.cn (S.K.); 215400000023@stu.haust.edu.cn (L.M.)

**Keywords:** Parkinson’s disease, camera sensor, video-based detection, attention, Mamba-2

## Abstract

The annual increase in the incidence of Parkinson’s disease (PD) underscores the critical need for effective detection methods and devices. Gait video features based on camera sensors, as a crucial biomarker for PD, are well-suited for detection and show promise for the development of portable devices. Consequently, we developed a single-step segmentation method based on Savitzky–Golay (SG) filtering and a sliding window peak selection function, along with a Cross-Attention Fusion with Mamba-2 and Self-Attention Network (CMSA-Net). Additionally, we introduced a loss function based on Maximum Mean Discrepancy (MMD) to further enhance the fusion process. We evaluated our method on a dual-view gait video dataset that we collected in collaboration with a hospital, comprising 304 healthy control (HC) samples and 84 PD samples, achieving an accuracy of 89.10% and an F1-score of 81.11%, thereby attaining the best detection performance compared with other methods. Based on these methodologies, we designed a simple and user-friendly portable PD detection device. The device is equipped with various operating modes—including single-view, dual-view, and prior information correction—which enable it to adapt to diverse environments, such as residential and elder care settings, thereby demonstrating strong practical applicability.

## 1. Introduction

Parkinson’s disease (PD) is the second most common neurodegenerative disorder worldwide, and its incidence has been steadily increasing in recent years [1]. Parkinson’s disease can lead to motor dysfunction and non-motor symptoms, such as tremors, rigidity, bradykinesia, and cognitive impairments, significantly impacting patients’ quality of life [2]. Given this, early diagnosis and timely intervention in PD are critically important for delaying disease progression [3,4,5]. However, due to insufficient public awareness of the condition, many patients fail to seek prompt medical attention at the onset of symptoms, thereby missing the optimal window for treatment. Therefore, the development of a PD detection method and portable device capable of rapid screening and auxiliary diagnosis holds significant practical value.

Common detection methods for PD include clinical assessment [6] and neuroimaging techniques [7]. However, these methods are relatively cumbersome and not conducive to the development of portable devices. Since PD is often accompanied by motor impairments, movement-related information also serves as a critical biomarker for PD diagnosis [8]. Gait—being the most common expression of human movement—naturally manifests without the need for professional guidance and effectively distinguishes PD patients from healthy individuals, making it an excellent biomarker [9]. In recent years, the rapid development of artificial intelligence has further expanded the application of data-driven gait analysis methods in PD detection, ushering in new advancements in this research field [10].

Gait can be digitized using both sensor-based and video-based methods. For instance, Biase et al. employed sensors to convert gait into kinetic data for gait analysis [11]. Shi et al. proposed an adaptive step detection algorithm based on sensor data [12], while Lancini et al. developed a biomechanics-based sensor gait recognition system that enables relatively effective gait detection [13]. Zhou et al. analyzed gait features using a single inertial sensor [14], and Shi et al. further introduced a deep learning-based inertial sensor model for gait detection [15]. Additionally, Alazeb et al. investigated the use of multi-sensor fusion for Parkinson’s disease detection [16]. These studies have provided valuable insights into gait mechanics and abnormal gait recognition. However, compared to the high cost and cumbersome operation of sensors, video data have recently gained greater favor among researchers due to their convenience and ease of acquisition. For example, Guo et al. utilized gait videos to extract skeletal joint data, subsequently employing a two-stream spatial–temporal attention graph convolutional network (2s-ST-AGCN) for PD detection [17]. And Zeng et al. combined skeletal joint data with silhouette data to construct a skeleton–silhouette fusion spatial–temporal graph convolutional network (ST-GCN) and Visual Geometry Group (VGG) network for assessing PD gait impairments [18].

However, it is noteworthy that most video-based data collection methods commonly face three issues: First, many data collections are limited to a single frontal or lateral view, lacking data from multiple views and related research. Second, the data collection process is relatively complex, relying on guidance from professionals and requiring substantial manual intervention during data processing, which poses significant obstacles to the development of portable devices for use by non-professionals. Third, the methods used in the aforementioned studies have not adequately considered the temporal characteristics of gait data, leaving room for further improvement.

To address the aforementioned issues, we propose an innovative solution. Firstly, our data collection encompasses both frontal and lateral views, providing a richer dataset for subsequent fusion. Based on this, we introduce the Cross-Attention Fusion with Mamba-2 and Self-Attention Network (CMSA-Net). CMSA-Net leverages the efficient time-series processing capabilities of Mamba-2 and self-attention to extract features from dual-view data, followed by cross-attention to effectively fuse frontal and lateral data. Additionally, we apply Maximum Mean Discrepancy (MMD) loss to ensure similarity in data distribution, thereby enhancing the overall fusion efficacy. To automatically extract single-step gait information from skeletal sequences, we propose the single-step segmentation method, which leverages Savitzky–Golay (SG) filtering and a sliding window peak selection function. Furthermore, we analyze the impact of frontal- and lateral-view data on PD detection, as well as the influence of prior information such as age and sex on the detection outcomes. Building upon the aforementioned methods, we develop a portable detection device characterized by its user-friendly operation and efficient data acquisition and processing workflows. This device offers a practical solution for the early screening and auxiliary diagnosis of PD, holding significant potential for clinical translation and application.

The main contributions of this study can be summarized as follows:

- To the best of our knowledge, we are the first to construct a full-body synchronized dual-view PD gait video dataset, which includes 304 HC samples and 84 PD samples, with a relatively large sample size.
- Based on the dual-view camera sensors of the data and characteristics of the skeletal sequence gait continuity, we propose a single-step segmentation method. Furthermore, we introduce CMSA-Net, which effectively processes temporal dual-view data and performs efficient information fusion, and employ MMD Loss to enhance the effectiveness of this fusion. In comparative analyses with multiple methods, our method achieved optimal results.
- Based on the aforementioned methods, we construct a portable device that not only achieves favorable detection performance but also exhibits excellent usability, thereby possessing strong practical significance.

The remainder of this paper is organized as follows: Section 2 reviews related work on PD detection using video-based gait data. Section 3 introduces the proposed method. Section 4 presents the experiments and results, providing both qualitative and quantitative analyses. Section 5 discusses the influence of camera sensor view and prior information on the results, the construction and usage of the device, and a comparative analysis with different studies. Finally, Section 6 concludes this study.

## 2. Related Work

In research on PD detection using gait videos, studies can be categorized based on data acquisition methods and classification schemes. First, video views can be classified into three types: frontal [17,19], lateral [18,20], and a combination of frontal and lateral [21]. Second, classification schemes can be divided into binary [19,21], three-class [18], and four-class [17,20] setups. In the binary class, the objective is to distinguish between PD and healthy control (HC) cases; in the four-class setup, classification is based on the Hoehn–Yahr stage [22] (with stages 3 and 4 merged into a single class); and the three-class method relies on ratings provided by professional clinicians [18].

Most studies employ a single view for data collection, but relying on a single view has a limitation because no study currently supports the optimality of any one side, and comparative experiments cannot be performed. Both Kaur et al.’s study and our work adopt a combined frontal and lateral view [21]. In Kaur et al.’s research, the videos recorded only the feet and required subjects to use a treadmill, whereas our study captures full-body gait videos with minimal equipment dependency, thereby enhancing practical applicability [21].

After processing, gait video data are typically represented as skeletal joint data. Consequently, some studies have adopted graph convolutional network (GCN)-based [23] methods. For instance, Zeng et al. employed ST-GCN [24], and Guo et al. used 2s-ST-AGCN [17]. Convolutional neural networks (CNNs) [19] have also demonstrated their utility in handling image data; for example, Ciresan et al. reported that a CNN achieved superior performance on a dataset [25], while Zeng et al. leveraged a VGG [26] architecture (improved CNN method) to process contour data. Furthermore, Transformer [27] architectures, known for their excellent sequence processing capabilities, have been applied to PD detection—for example, Endo et al. employed a Transformer-based method, GaitForeMer [20], for PD detection. In recent years, a number of other effective temporal data processing methods have emerged, such as iTransformer [28], Joint Time–Frequency Domain Transformer (JTFT) [29], and Mamba-2 [30], all of which have achieved state-of-the-art (SOTA) results in their respective fields. Based on the current research landscape and the characteristics of our data, we propose Cross-Attention Fusion with Mamba-2 and Self-Attention Network (CMSA-Net), which demonstrates superior performance compared to other methods in our experiments.

## 3. Data and Methods

### 3.1. Data

Data for our study were collected through a collaborative project with the First Affiliated Hospital of Henan University of Science and Technology. From 7 June 2023 to 11 September 2024, we collected video gait data from 304 HC (healthy control) volunteers and 84 PD (Parkinson’s disease) volunteers. The health status of all volunteers was assessed and confirmed by PD-related professional doctors.

**Figure 1:**
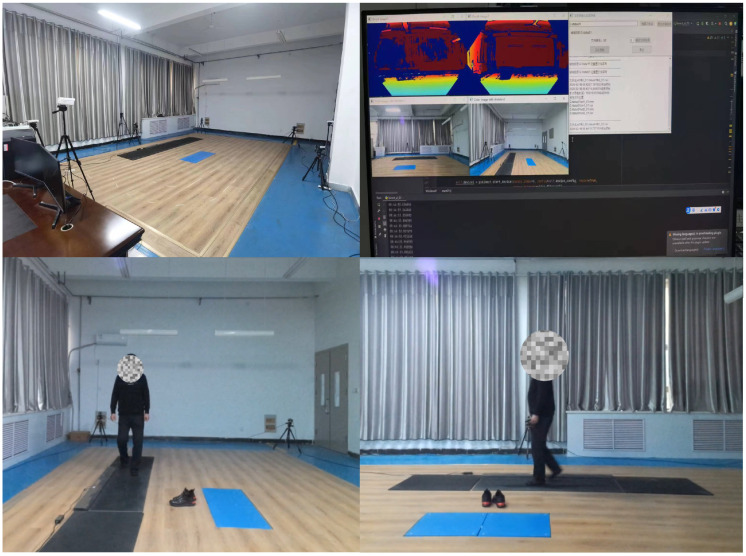
Data collection environment.

We used two camera sensors to synchronously record gait videos—one positioned frontally and the other laterally—with a resolution of 1080p at 30 frames per second. Each volunteer participated in approximately 5 min of gait recording and was instructed to walk back and forth within the camera sensor coverage area, with separate video recordings made at both 4 m and 2 m distances from the lateral camera sensor. Volunteers maintained a natural walking gait, walking straight and perpendicular to the frontal camera sensor, without running, jumping, or other unnatural walking behaviors. The data collection environment is shown in Figure 1.

All experimental procedures were approved by the Ethics Committee of the First Affiliated Hospital of Henan University of Science and Technology (Approval No. 2023-03-K0029), and written informed consent was obtained from all volunteers. Table 1 presents the demographic characteristics of the volunteers.

### 3.2. Data Processing

The raw dual-view videos were preprocessed through three key steps: skeletal joint extraction, single-step segmentation, and standardization, as detailed below.

#### 3.2.1. Skeletal Joint Representations

**Figure 2:**
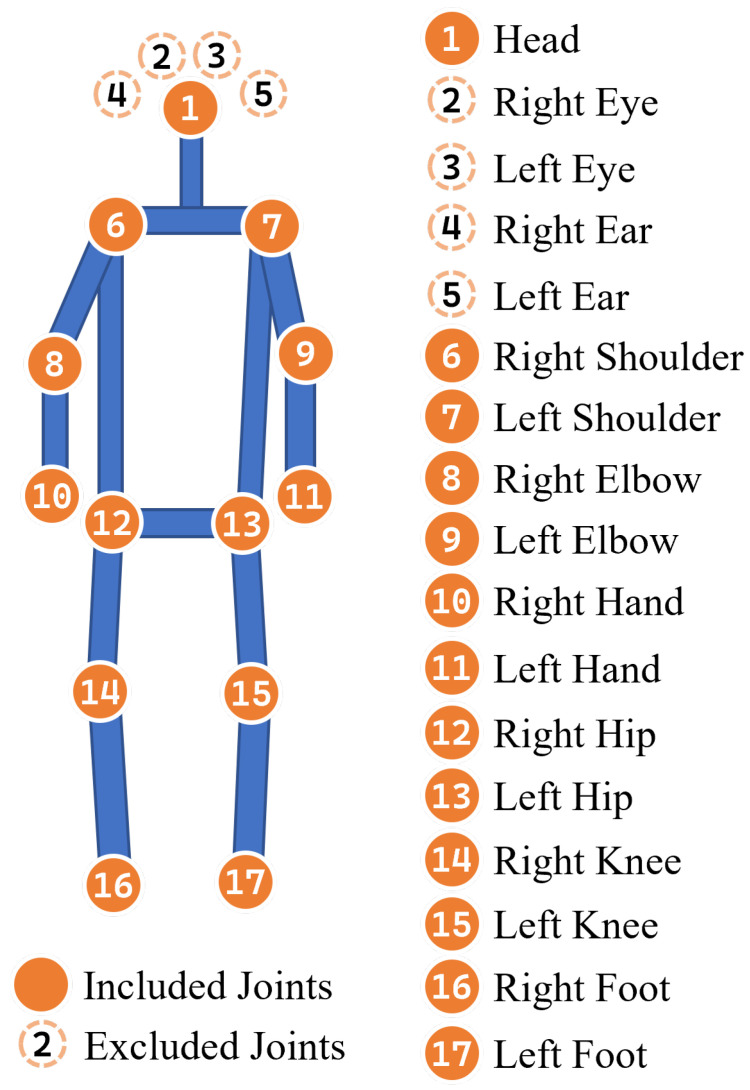
Skeletal joint topology.

In gait detection research, converting human motion videos into skeletal joint representations is an effective method for data processing. These skeletal representations not only reduce data complexity but also preserve the essential motion patterns of the human body. We used YOLOv7-Pose [31] to process gait videos and obtain skeletal joint data, which included 17 joints, as shown in Figure 2. Non-essential joints, such as the eyes and ears, were excluded, yielding 13 joints for subsequent analysis.

#### 3.2.2. Single-Step Segmentation Method

The processed skeletal joint data comprised continuous back-and-forth walking sequences with a relatively long duration, which could not be directly utilized for training and thus required appropriate preprocessing. Single-step sequencing effectively captures the gait pattern characteristics of different samples, making it an optimal input format. Moreover, automatically extracting single-step sequences from complex long videos aligns with the core research objective of device development, which emphasizes minimizing operational demands on users.

We define a single-step as the sequence from one instance of the right foot lifting and landing to one instance of the left foot lifting and landing. Single-step sequences can be extracted by leveraging the inherent properties of skeletal joint data, which exhibit periodic changes with gait motion, particularly in the feet. To extract single-step sequences, it is necessary to obtain the frames for the right foot’s landing, as well as the corresponding frames for the left foot, which serve as the segmentation frames. The Y-axis data from the lateral view clearly reflects the distance between the foot and the ground, with the local minimum of this distance corresponding to the landing frames.

We developed a computational method that integrates Savitzky–Golay (SG) filtering [32] with a sliding window peak selection function to obtain segmentation frames. This method processes skeletal joint data, focusing on the lateral-view data in relation to the Y-axis positions of the left and right foot lift heights. The goal is to obtain the local minimum of the right foot’s Y-coordinate as the start frame and the left foot’s as the end frame.

**Figure 3:**
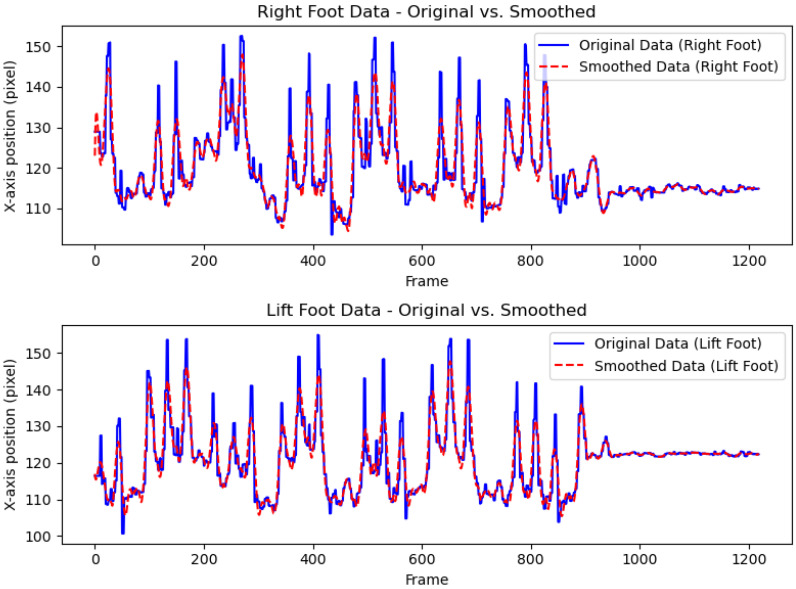
Comparison of original and SG-filtered foot data.

Therefore, we focused on the positions of local minima, which can be affected by noise. To address this, we used an SG filter to reduce noise while preserving extrema positions. Figure 3 illustrates an example of lateral-view data variations before and after applying the SG filter.

To robustly detect these minima, we employed a sliding window peak selection function. This function operates by traversing the data sequence with a fixed-width window, systematically analyzing local extrema within each window. By adjusting key parameters, including window size, minimum peak separation, and peak prominence threshold, the desired local maximum can be extracted while avoiding the selection of stable regions in the curve. In practice, negating the target curve allows the peak selection function to extract local minima.

**Figure 4:**
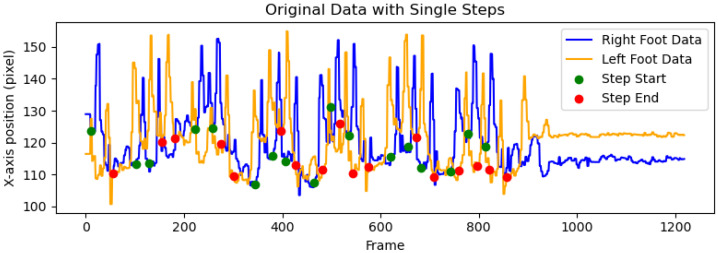
Single-step segmentation.

In Figure 4, the X-coordinates of Step Start (marked in green) and Step End (marked in red) represent the segmentation frames. Each Step Start corresponds uniquely to a Step End, and the interval between them is considered a single step. Between these two points, one complete right foot lift and landing (blue curve) followed by one complete left foot lift and landing (yellow curve) occur. Additionally, the periods when the volunteer remained stationary were not included in the analysis.

The aforementioned data primarily represent lateral-view data, while the frontal-view data are temporally aligned with the lateral view. Therefore, the same segmentation frame can be applied to both.

Our device is designed for PD detection, with older adults as its primary target users. Strict usage requirements, such as precise start and end times and continuous gait, are impractical as they undermine the device’s practicality. Our proposed method reduces ineffective and harmful inputs while avoiding complex usage requirements, thereby enhancing both device performance and user experience.

#### 3.2.3. Standardization

Considering the application of the device, the goal is to standardize skeletal joint data captured at different distances into a similar scale. This allows users to operate the device over a broader range of distances without strict placement constraints.

We scale the skeletal joint data so that the *x*-coordinates have a mean of 0, and the *y*-coordinates fall within the range of 0 to 1, while maintaining the aspect ratio. Let the *x*-coordinate data be ${\left\{x_{i}\right\}}_{i=1}^{N}\in R$ and the *y*-coordinate data be ${\left\{y_{i}\right\}}_{i=1}^{N}\in R$. The standardization formulas are as follows:

(1) $$x_{i}^{\prime }=\frac{x_{i}-\mathrm{mean}\left(x\right)}{\max(y)-\min(y)}$$

(2) $$y_{i}^{\prime }=\frac{y_{i}-\min\left(y\right)}{\max(y)-\min(y)}$$

Each single-step sequence has a variable frame length, with an average of approximately 50 frames. To standardize the input length for model training, we adjust all steps to 50 frames by either trimming or applying linear interpolation.

Given a set of coordinates, ${\left\{x_{i},y_{i}\right\}}_{i=1}^{N}$, linear interpolation is performed using the following formula:

(3) $$y_{\mathrm{new}}=y_{i}+\frac{y_{i+1}-y_{i}}{x_{i+1}-x_{i}}\left(x_{\mathrm{new}}-x_{i}\right)$$

Here, $x_{\mathrm{new}}$ denotes the desired interpolated position, determined by the number of target frames.

Finally, Z-score normalization is applied to the entire processed dataset to further standardize the distribution:

(4) $$z_{i}=\frac{x_{i}-\mu }{\sigma }$$

where μ is the mean and σ is the standard deviation of the sequence.

### 3.3. CMSA-Net Architecture

**Figure 5:**
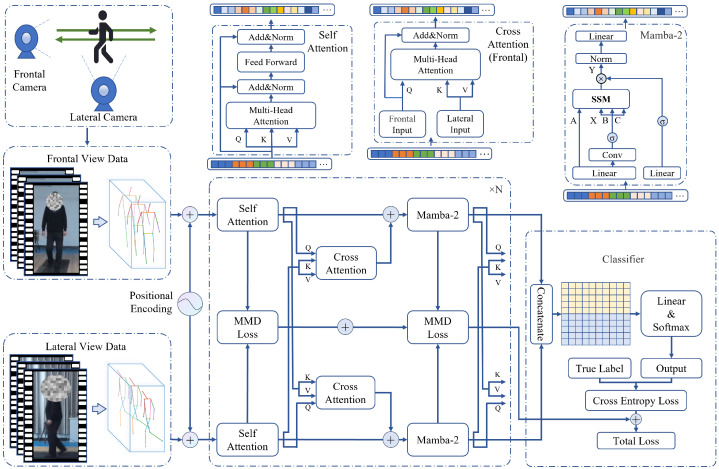
Cross-Attention Fusion with Mamba-2 and Self-Attention Network.

The overall pipeline of our proposed Cross-Attention Fusion with Mamba-2 and Self-Attention Network (CMSA-Net) is shown in Figure 5. The input consists of gait skeleton joint sequences captured from both frontal and lateral views. These two sequences are processed separately by identical networks composed of multiple layers of Self-Attention and Mamba-2 modules to extract their respective features.

Since both inputs represent different views of the same gait, information sharing between the two networks is facilitated through cross-attention in each layer. The extracted features from both streams are concatenated and fed into a classifier consisting of a linear layer followed by a softmax function to obtain the final detection results.

Given that the information distributions of the two inputs are similar, the Maximum Mean Discrepancy (MMD) [33] is employed to measure their discrepancy, with MMD loss serving as a regularization term. During training, the total loss consists of MMD loss and cross-entropy loss.

Compared to using attention or Mamba-2 alone, our proposed network combines the advantages of both approaches, achieving a higher training speed while maintaining competitive accuracy. Additionally, the use of cross-attention between the two input streams provides an efficient mechanism for feature interaction, enabling the network to capture complementary information without significantly increasing computational complexity. The introduction of the MMD loss further enhances feature fusion without incurring additional computational costs, ensuring a balanced trade-off between accuracy and efficiency.

#### 3.3.1. Temporal Feature Extraction Modules

**Attention:** Attention, as a fundamental component of the Transformer architecture, has been widely adopted and is particularly well-suited for handling time-series problems. The core mechanism of Attention uses Query (Q), Key (K), and Value (V) vectors for weighted learning. In the context of the skeletal joint time series discussed in this paper, it enables the assignment of higher weights to frames with more distinctive features within continuous gait sequences, thereby facilitating more efficient information processing.

For the self-attention mechanism, given a sequence, $X={\left\{x_{i}\right\}}_{i=1}^{N}\in R^{n\times d}$, where *n* denotes the sequence length and *d* represents the embedding dimension, the computation proceeds as follows:

(5) $$Q=XW_{Q},\;K=XW_{K},\;V=XW_{V}$$

where $W_{Q},W_{K},$ and $W_{V}$ are learnable weight matrices.

In contrast, the proposed cross-attention framework for fusing frontal- and lateral-view data computes the QKV matrices differently. Let $X={\left\{x_{i}\right\}}_{i=1}^{N}\in R^{n\times d}$ denote the frontal-view data and $Y={\left\{y_{i}\right\}}_{i=1}^{N}\in R^{n\times d}$ represent the lateral-view data. The frontal-view QKV is computed as

(6) $$Q=XW_{Q},\;K=YW_{K},\;V=YW_{V}$$

while the lateral-view QKV output is computed as

(7) $$Q=YW_{Q},\;K=XW_{K},\;V=XW_{V}$$

The computation method of Attention is

(8) $$\mathrm{Attention}\left(Q,K,V\right)=\mathrm{softmax}\left(\frac{QK^{T}}{\sqrt{d_{k}}}\right)V$$

where $\sqrt{d_{k}}$ serves as a scaling factor to stabilize gradient propagation.

Practically, the complete Attention block consists of multi-head attention, feed-forward networks (FFNs), and layer normalization. The complete Attention block is depicted in Figure 5.

**Mamba-2:** Mamba-2 is a novel network architecture based on state space models (SSMs) and Structured State Space Duality (SSD) for constructing computational modules. It has been shown to outperform Transformers on several datasets and operates more than twice as fast. A paper on Mamba-2 indicates that combining Mamba-2 layers with Attention layers yields better results [30]. Therefore, we adopt an alternating arrangement of Attention and Mamba-2 layers to construct the computational module.

The SSM formulation defines a discrete-time dynamical system by

(9) $$h_{t}=Ah_{t-1}+Bx_{t},$$

(10) $$y_{t}=Ch_{t}$$

where A,B, and *C* are learnable weight matrices, $x_{t}$ is the input ${\left\{x_{i}\right\}}_{i=1}^{N}\in R$ at time *t*, $h_{t}$ is the hidden state, and $y_{t}$ is the output ${\left\{y_{i}\right\}}_{i=1}^{N}\in R$ at time *t*.

Recursively unrolling $h_{t}$ gives

(11) $$\begin{array}{cc} h_{t} & =A_{t}\cdots A_{1}B_{0}x_{0}+A_{t}\cdots A_{2}B_{1}x_{1}+\cdots +A_{t}A_{t-1}B_{t-2}x_{t-2}\\ \; & \;+A_{t}B_{t-1}x_{t-1}+B_{t}x_{t}\\ \; & =\sum_{s=0}^{t}\left(\prod_{i=s}^{t}A_{i}\right)B_{s}x_{s} \end{array}$$

$y_{t}$ can be expressed as

(12) $$y_{t}=C_{t}^{⊤}\sum_{s=0}^{t}A_{t:s}^{\times }B_{s}x_{s}$$

The output *y* can be represented by simplification as a function of A,B, and *C* and the input *x*:

(13) y=SSM(A,B,C)(x)=Mx

(14) $$M_{ji}:=C_{j}^{⊤}\left(\prod_{k=i+1}^{j}A_{k}\right)B_{i}$$

It can be observed that the rank below the diagonal of matrix *M* is full, indicating that it is a semi-separable matrix. In Mamba-2, the SSD algorithm simplifies the computation of semi-separable matrices, thereby streamlining the calculation of *M*. It achieves this by partitioning and converting them into low-rank matrices.

In practical application, a Mamba-2 module is composed of several parts, as shown in Figure 5. Specifically, Conv represents the convolutional layer, Linear represents the linear layer, and σ represents the nonlinear activation function.

#### 3.3.2. Maximum Mean Discrepancy-Based Loss

CMSA-Net facilitates inter-layer frontal and lateral information exchange via a cross-attention block. Given that the frontal- and lateral-view sequences represent multi-view observations of the same gait cycle, they share informational similarities. We aim to progressively strengthen this similarity during the training process to achieve better feature fusion. To achieve this goal, we propose a loss regularization term based on MMD to regulate the training process.

MMD can describe the distribution similarity between two datasets. The smaller the MMD is, the more similar the feature distributions of the two inputs are. Suppose that the data for the frontal view are $X={\left\{x_{i}\right\}}_{i=1}^{N}\in R$ and for the lateral view are $Y={\left\{y_{i}\right\}}_{i=1}^{N}\in R$; the formula for calculating MMD is

(15) $$D^{2}=\frac{1}{n(n-1)}\sum_{i\neq j}K\left(x_{i},x_{j}\right)+\frac{1}{m(m-1)}\sum_{k\neq l}K\left(y_{k},y_{l}\right)-\frac{2}{nm}\sum_{i=1}^{n}\sum_{k=1}^{m}K\left(x_{i},y_{k}\right)$$

where $K\left(x,y\right)=\exp(-\gamma ∥x-y{∥}^{2})$ is a Gaussian kernel function, and ${∥x-y∥}_{2}=\sqrt{\sum_{k=1}^{d}{\left(x_{k}-y_{k}\right)}^{2}}$ is the Euclidean distance. γ is the bandwidth function of the kernel function, and the calculation method is

(16) $$\gamma =\frac{1}{(\mathrm{median}\{∥Z_{i}-Z_{j}{∥}_{2}|1\leq i<j\leq n+m{\})}^{2}}$$

where $Z=\left[\begin{array}{c} X\\ Y \end{array}\right]$.

For *l* layers of Attention or Mamba-2, the MMD loss $L_{d}$ is

(17) $$L_{d}=\frac{1}{l}\sum_{i=1}^{l}D_{i}$$

The total loss includes not only MMD loss, $L_{d}$, but also cross-entropy loss and the regularization term. Due to class imbalance, weighted cross-entropy loss is used to correct for this. For *N* samples, $L_{w}$ can be expressed as

(18) $$L_{w}=-\sum_{i=1}^{N}\left(w_{1}y_{i}\log\left(p_{i}\right)+w_{2}\left(1-y_{i}\right)\log\left(1-p_{i}\right)\right)$$

where $w_{1}$ and $w_{2}$ are the class weights, $p_{i}$ is the predicted probability, and $y_{i}$ is the true label.

We add L1 regularization to sparse weights and L2 regularization to prevent overfitting:

(19) $${∥W∥}_{1}=\sum_{j}\left|w_{j}\right|$$

(20) $${∥W∥}_{2}^{2}=\sum_{j}w_{j}^{2}$$

The total loss can be expressed as

(21) $$L=L_{w}+\lambda_{1}L_{d}+\lambda_{2}{∥W∥}_{1}+\lambda_{3}{∥W∥}_{2}^{2}$$

where $\lambda_{1},\lambda_{2},\lambda_{3}$ are hyperparameters in training that adjust the weights of $L_{d}{,∥W∥}_{1},{∥W∥}_{2}^{2}$ in the total loss function.

## 4. Experiments and Results

### 4.1. Dataset and Evaluation Metrics

After single-step segmentation, the HC single-step dataset comprised 26,999 samples, and the PD single-step dataset comprised 11,472 samples, with a sample ratio of 0.72:0.28, totaling 37,471 samples. Each dataset point consisted of a matrix of the size (1, 50, 39), where 50 was the number of frames after linear interpolation, and 39 was the combination of 13 coordinate points with their x-axis data, y-axis data, and joint confidence scores. The dataset was standardized so that the mean was 0 and the standard deviation was 1.

The experiment used five-fold cross-validation (CV), where the total dataset was divided into five equal parts, four of which were used for training and one for validation. The training set and validation set did not contain data from the same individual. Five experiments were conducted using the same parameters and random seed. The results presented are the average values from five-fold CV, unless otherwise specified.

The results from the five-fold CV provide a comprehensive evaluation of the method’s performance across different subsets of the data and utilize the following evaluation metrics:

**Accuracy (Acc)** measures the proportion of correctly predicted samples.

(22) $$\mathrm{Acc}=\frac{TP+TN}{TP+TN+FP+FN}$$

where TP is True Positives, TN is True Negatives, FP is False Positives, and FN is False Negatives.

**Precision (Prec)** indicates the ratio of true positive instances among all samples predicted as positive.

(23) $$\mathrm{Prec}=\frac{TP}{TP+FP}$$

**Recall (Rec)** evaluates the method’s ability to correctly identify positive instances from all actual positive samples.

(24) $$\mathrm{Rec}=\frac{TP}{TP+FN}$$

**F1-score (F1)** is the harmonic mean of precision and recall, providing a balanced measure of both metrics’ performance.

(25) $$F1=2\times \frac{\mathrm{Precision}\times \mathrm{Recall}}{\mathrm{Precision}+\mathrm{Recall}}$$

Additionally, the Area Under the Curve (AUC) of the Receiver Operating Characteristic (ROC) curve reflects the overall performance of the method by depicting the relationship between the false positive rate and true positive rate across different thresholds. A higher AUC value indicates stronger classification ability of the method.

### 4.2. Implementation Details

Our network was implemented in Python 3.8 using common deep learning libraries such as PyTorch 2.0.1, Scikit-Learn 1.2.2, and NumPy 1.23.5. The experiments were conducted on a computer equipped with an Intel i9-13900K CPU and a RTX 4090 GPU with 32 GB of memory. The training batch size for all experiments was 3072, with 1000 epochs.

The training was performed using the AdamW [34] optimizer with the parameters $\beta_{1}=0.9$ and $\beta_{2}=0.99$. Warm-up [35] and learning rate decay strategies were applied. During the first 200 epochs, the learning rate gradually increased from $1\times 10^{-8}$ to $1\times 10^{-4}$. In the subsequent 400 epochs, the learning rate remained constant, and in the final 400 epochs, the learning rate gradually decayed to $1\times 10^{-6}$. The model was saved when it achieved the highest F1-score.

Through testing, we found that a four-layer network depth with alternating self-attention and Mamba-2 achieved the best results. The remaining parameters used throughout the entire experimental process, including data processing, are presented in Table 2, with all parameters having been optimized.

### 4.3. Experiment and Analysis

#### 4.3.1. Classification Result

The experimental results obtained from the five-fold CV are presented in Table 3. Our proposed method achieved an Acc of 89.1% and an F1 of 81.1%. The average AUC was 0.928.

#### 4.3.2. Comparison with Different Methods

To validate the effectiveness of our proposed method, we conducted comparison experiments with several classical methods and advanced time-series- or joint-based methods (see Table 4). Our method outperformed these methods, achieving the highest Acc, F1, and AUC. Compared to the advanced time-series method Joint Time–Frequency Domain Transformer (JTFT), our method achieved a 1.0% higher Acc, 1.3% higher F1, and 0.012 higher AUC, along with a 3.3% higher Prec. Our method also performed well compared to common joint-based methods such as GCN, ST-GCN, and 2s-ST-GCN. Although iTransformer excelled in Rec, its overall performance was not as superior as that of our method.

Overall, although our method shows some limitations in recall compared to iTransformer and JTFT, it surpasses the existing methods in terms of Acc, Prec, F1, and AUC, suggesting promising overall performance.

#### 4.3.3. Ablation Experiments

To analyze the impact of different methods on the overall results, we conducted ablation experiments, with the results presented in Table 5. Data Parallelism indicates whether dual-channel processing is applied to data from bilateral camera sensors. Through ablation experiments, it was observed that the baseline Attention achieved an Acc of 87.4% and an F1 of 78.0%. With the introduction of Data Parallelism, Acc increased by 0.6% and F1 improved by 1.6%. When cross-attention was applied for information fusion, Acc improved by 0.9% and F1 by 1.2%. Replacing part of the Attention modules with Mamba-2 resulted in an increase of 0.2% in Acc and 0.4% in F1.

Although the improvement from substituting Attention with Mamba-2 is relatively modest, the computational speed of Mamba-2 exceeds that of Attention, making it produce performance gain with negligible cost. Overall, the ablation experiments showed that each modification led to improvements in Acc, F1, and AUC.

#### 4.3.4. Loss Parameters Experiments

The proposed MMD loss parameter was evaluated through comparative experiments, as shown in Table 6. When $\lambda_{1}=0.01$, the Acc improved by 0.7% and the F1 by 0.8% compared to those when not using MMD Loss ($\lambda_{1}=0$). However, when $\lambda_{1}=0.1$, the constraints were too strict, leading to a decline in overall performance.

Due to sample imbalance (HC samples ∼ 70%, PD samples ∼ 30%), we attempted to address this by adjusting the Weighted Cross-Entropy Loss parameters, $w_{1}$ and $w_{2}$, as shown in Table 7. We first set the compensation ratio to match the sample imbalance ratio, $w_{1}=0.6$, $w_{2}=1.4$, but this method yielded poor results. Although *Rec* improved, other metrics significantly declined, resulting in an *Acc* of only 85.6% and an *F1* of 77.4%.

By optimizing, we obtained $w_{1}=0.9$ and $w_{2}=1.1$. Compared to no imbalance compensation, with $w_{1}=1.0$ and $w_{2}=1.0$, this adjustment led to a 0.7% increase in Acc and a 0.5% improvement in the F1. Overall, the proposed loss function method was proven to be effective, and it necessitates certain parameter optimization.

## 5. Discussion

### 5.1. Comparative Study of Frontal and Lateral View

Different studies have collected PD video data using various methods, typically focusing on frontal and lateral views. In contrast, our dataset includes both views from the same individual, simultaneously captured, allowing for an equal comparison of their impact on the results. To conduct a comparative analysis of these views, we performed experiments using the same single-side network, and the results are presented in Table 8.

Table 8 shows that the lateral view outperforms the frontal view in Acc, Prec, and F1, with a slightly higher AUC, while the frontal view has a higher Rec. If used solely as a disease detection method, a high recall rate is crucial, as it helps reduce the risk of missing PD patients. However, for a home-use portable device, high precision is crucial, as false positives can lead to unnecessary costs, leading to wasted time and effort for family members taking elderly individuals to medical appointments. Therefore, in terms of the combined metrics of Acc and F1, the lateral view outperforms the frontal view.

Considering the application of the device, the lateral view allows for single-step segmentation through the motion curve of the foot’s skeletal joints. However, due to perspective, single-step segmentation in the frontal view becomes much more complex and less accurate. Therefore, from an application view, if only one view is used, the lateral view is preferable.

Overall, both in terms of results and application, the lateral view outperforms the frontal view. However, the data from the frontal and lateral views exhibit complementarity, and combining both views leads to improvements in overall performance.

### 5.2. Fine-Tuning Based on Prior Information

We collected physiological prior information on sex, age, height, weight, BMI, shoe size, and single-step duration for all volunteers, which could be used to improve the method’s performance and analyze the relationship between these priors and PD. Specifically, all prior information was first Z-score-standardized and then passed through an unbiased Linear layer, and the resulting adjustment was added to the original softmax output, followed by a final Softmax layer. During the training process, the original method was kept frozen, and only the newly added Linear layer was fine-tuned. A total of 500 epochs were used for fine-tuning, and the results are shown in Table 9.

In Table 9, it is shown that fine-tuning with prior information improved the method by 0.6% in Acc and 0.7% in F1, although the AUC decreased. However, this is entirely reasonable, as the adjustment from the prior information made the classification boundary relatively fuzzier, which could lead to a decrease in the AUC. Nonetheless, fine-tuning with prior information improved the overall performance of the method.

**Figure 6:**
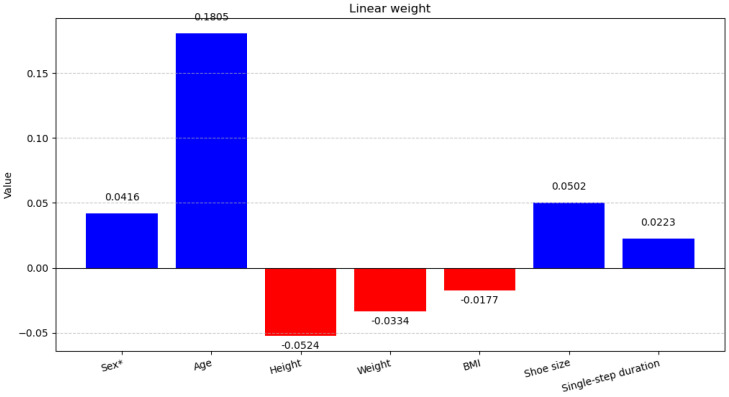
Linear weight with prior information. * The prior information data uses 1 to represent male and −1 to represent female.

Because we used an unbiased Linear layer to obtain the prior information adjustment, we can analyze the impact of different prior information data on the classification result through the weights of this layer. Figure 6 shows all the prior information used for training and the magnitude of the weights in the Linear layer that affect PD, where positive values indicate a positive impact (blue in the figure), meaning a higher likelihood of PD, and negative values indicate a negative impact (red in the figure). By analyzing the literature, we explore the practical significance of the prior information on Linear weight in Figure 6.

The Sex weight in the Linear weight layer is 0.0416, indicating a slight increase in the probability of PD in males. This finding aligns with the sex-related conclusions in the review study by Zhu et al. [38], while studies by Terrin et al. [39] and Chen et al. [40] further analyze the underlying mechanisms. Overall, most studies agree that males are more susceptible to PD. However, according to Dumas et al. [41], sex itself induces biomechanical differences in movement patterns, and how such differences influence PD gait characteristics warrants further investigation.

The Age weight of 0.1805 indicates a strong positive correlation between age and the probability of developing PD, which is a well-established fact. This has been extensively validated by numerous studies and will not be further elaborated here.

Research on the relationship between PD and height is relatively scarce. An early study by Ragonese et al. [42] found a negative correlation between height and PD in males, while no such association was observed in females. Similarly, a study by Saari et al. [43], based on autopsy analysis of PD samples, suggests that shorter stature may be associated with a lower number of dopaminergic neurons, potentially increasing the risk of PD. These studies provide some evidence for Height weight of −0.0524.

Weight loss is a common clinical manifestation of PD [44,45], which can explain the Weight weight of −0.0334.

The relationship between BMI and PD has been extensively studied with intriguing findings. In a female-exclusive study by Portugal et al. [46], women with higher BMI showed lower PD incidence. Conversely, a study by Kim et al. [47] indicates that individuals with lower BMI have higher PD risk, while overweight status shows no association with PD. Studies by Palacios et al. [48] and Wang et al. [49] found no significant association between BMI and PD prevalence. However, in a male-specific study by Osler et al. [50], higher BMI in men correlated with increased PD risk. As a composite measure of height and weight, BMI’s influence appears complex. Our result, with a BMI weight of −0.0177, represents a relatively low negative weight, which falls within a reasonable range.

There is almost no research on the relationship between shoe size and PD. We believe that male shoe sizes (approximately 40–43) and female shoe sizes (approximately 36–39) differ significantly, which may be attributed to sex differences. Therefore, the Shoe size weight (0.0502) may be considered as an indirect representation of the Sex weight (0.0416).

Although stride length and walking speed are clearly reduced in PD patients, the duration of a single step does not necessarily increase significantly. For example, in the study by Veer et al. [51], the difference in single-step duration between HC and PD was not significant, with PD having a slightly longer single-step duration on average than HC. This is consistent with the meaning of the slight positive weight observed in our Duration of single step weight (0.0223).

Overall, the weights obtained from the unbiased Linear layer based on prior information can be corroborated with existing research, and using prior information can effectively improve the method’s performance. However, we should not mandate that users must input this prior information in order to use the device. In the practical device, we incorporate prior information as an additional adjustment, allowing users to choose whether to use it.

### 5.3. Portable Device

**Figure 7:**
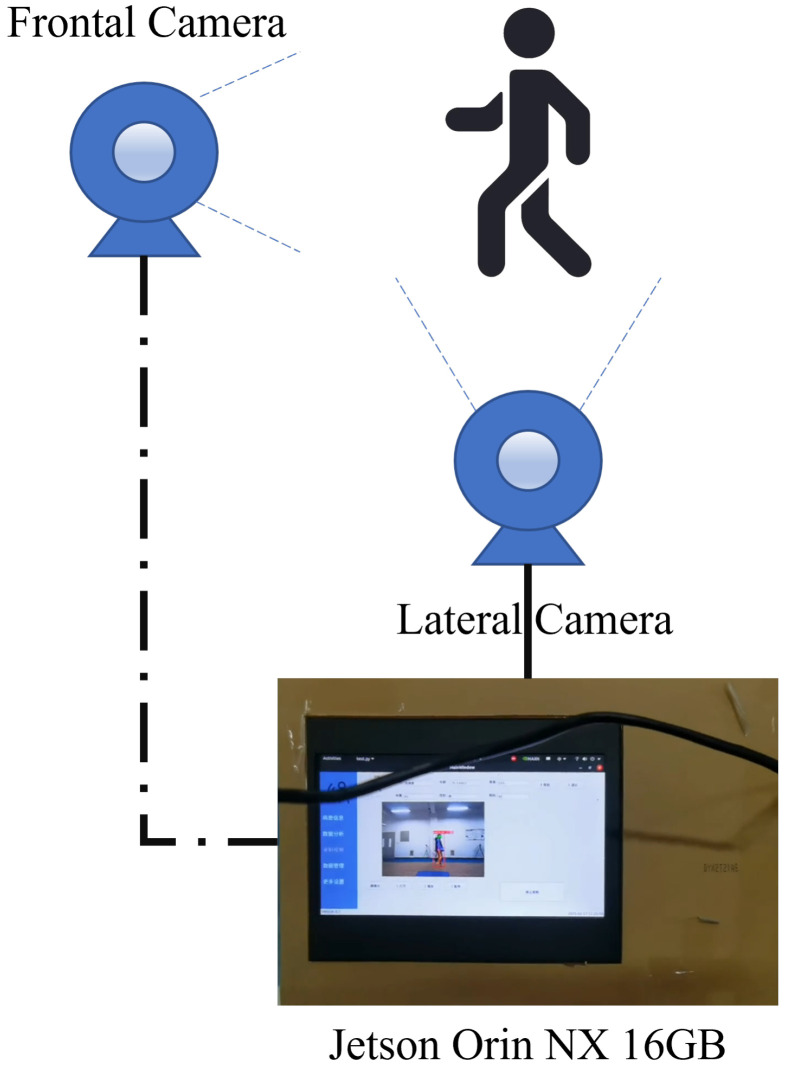
Portable device.

We developed a portable device based on CMSA-Net, with its core computing unit being the NVIDIA Jetson Orin NX, manufactured by NVIDIA Corporation, Santa Clara, CA, USA. The device can be configured to operate in either a single-camera (only lateral camera) or dual-camera mode, and it allows the optional entry of prior information about the test subject. Its structure is shown in Figure 7, and the metric results are presented in Table 10.

Different usage modes are designed to adapt to various environments. For example, in spacious nursing homes or senior activity centers, the device configured with both frontal and lateral camera sensors can be fixedly installed in public areas frequently used by older adults. After pressing the designated on-screen button, the individual simply walks back and forth within a specified area. When potential cases are detected, staff can assist by entering prior information to achieve more accurate results. For temporary or portable applications, using only the lateral camera sensor is sufficient, rendering the device similar to a smart phone that can perform detection when held steadily.

Overall, this portable device allows for flexible deployment in various environments, making it suitable for both institutional and individual use to facilitate PD detection. However, during device usage, participants are still required to walk in a relatively regular and straight manner. In future research, we plan to collect data on other types of trajectories, such as circular, diagonal, and irregular walking, to expand the device’s applicability and reduce its usage limitations.

### 5.4. Comparison with Present Studies

We compared studies in recent years that employed data and methods similar to those used in our research, with the results presented in Table 11. Due to variations in data processing methods, sample sizes, and sample proportions across studies, although the statistical metrics in the table are not directly comparable, they still offer some reference value.

It can be observed that our method achieved the highest Acc. Although the F1 was not the highest, this can be attributed to the difference in sample distribution. In He et al.’s study [19], the ratio of HC to PD samples was approximately 1:1, whereas in our study, the ratio was around 0.8:0.2. As a result, a slightly lower F1 is expected.

Kaur et al. [21] and we both utilized frontal- and lateral-view data. However, since Kaur et al. did not consider the fusion of bilateral data and relied solely on CNN for processing, their results were less optimal. In contrast, our data included all body joints and did not require additional equipment, resulting in better performance and practicality.

Our dataset includes the largest total number of participants. Although the number of PD samples is not the highest and there is an imbalance between HC and PD samples, this imbalance better reflects real-world scenarios, where the number of HC individuals is naturally higher than PD cases. Therefore, our results are more representative of practical applications.

The multi-class classification of PD also holds significant clinical value. In our current study, which primarily focuses on developing a user-friendly portable device, we adopted a binary classification method as the main outcome to facilitate user interpretation. In future research, we will utilize this dual-view dataset to explore multi-class classification of PD.

## 6. Conclusions

We collected a dual-view gait video dataset comprising 304 HC and 84 PD volunteers. Considering the characteristics of this dataset, we proposed a novel gait detection method, CMSA-Net. The method employs a cross-attention mechanism to integrate self-attention features from different views with Mamba-2 block features. Moreover, we introduced the MMD loss to optimize the distribution of features, thereby enhancing the fusion effectiveness. In comparative experiments with multiple methods, CMSA-Net achieved the best performance, demonstrating its effectiveness in PD gait detection tasks.

Furthermore, to facilitate the development of a portable PD detection device, we adopted a single-step segmentation method during data preprocessing. This method discards static states and extracts only effective gait segments, improving overall detection performance while enhancing ease of use. This improvement is particularly significant for a device primarily designed for elderly users. Additionally, we analyzed the impact of video viewpoints and prior information on method performance, providing valuable insights for future research.

Based on these methodologies, we developed a portable PD detection device that supports both single- and dual-view modes and offers optional integration of prior information. This flexibility enables adaptation to various application scenarios, such as home and elder care institutions, highlighting its practical applicability.

In future work, we will develop multi-class classification methods and devices for applications in hospitals and assistive healthcare settings, where more precise grading and assessment are required.

## Figures and Tables

**Table 1:** Demographic characteristics of volunteers (mean ± SD).

Characteristics	HC	PD
Participants (male/female)	304 (152/152)	84 (46/38)
Age (years)	55.8 ± 9.2	62.7 ± 9.7
Height (cm)	165.6 ± 7.2	165.3 ± 7.4
Weight (kg)	69.2 ± 11.9	66.3 ± 12.1
BMI * (kg/m^2^)	24.53 ± 3.15	24.43 ± 3.14
Hoehn–Yahr stage	N/A	1.68 ± 0.86

* The Body Mass Index (BMI) is a commonly used indicator to assess body weight in relation to height. BMI = Weight(kg)/(Height(m))^2^.

**Table 2:** All parameters used in the experiment.

Module	Parameter	Sub-Parameter	Value
Single-step segmentation	SG filter	Window size	45
		Polynomial order	6
	Peak selection function	Window size	30
		Minimum peak separation	30
		Peak prominence threshold	Mean(height) */20
Backbone network	Self-attention block	Maximum sequence length	50
		Embedding dimension	39
		Heads	3
		Feedforward dimension	64
		Dropout	0.2
	Cross-attention block	Maximum sequence length	50
		Embedding dimension	39
		Heads	3
	Mamba-2 block	Model dimension	39
		State dimension	32
		Convolution kernel size	1
		Expansion factor	1
		Heads	3
		Matrix partition size	10
		Dropout	0.2
Loss function		$w_{1}$	0.9
		$w_{2}$	1.1
		$\lambda_{1}$	0.01
		$\lambda_{2}$	0.0001
		$\lambda_{3}$	0.001

* Mean(height) is the average height of the samples in a video, where height is calculated as the difference between the Y-axis data of the head joint and the Y-axis data of the midpoint between the two foot joints.

**Table 3:** The results of 5-fold CV.

Fold	Training		Validation				
	**Acc%**	**F1%**	**Acc%**	**Prec%**	**Rec%**	**F1%**	**AUC**
1st Fold	96.8	94.5	89.3	85.0	77.6	81.1	0.940
2nd Fold	95.1	91.8	85.0	75.0	72.3	73.6	0.882
3rd Fold	96.5	94.0	89.0	86.1	76.3	80.9	0.916
4th Fold	95.8	92.7	92.2	86.1	87.7	86.9	0.968
5th Fold	97.5	95.6	90.1	86.7	79.6	83.0	0.935
**Mean**	96.3	93.7	89.1	83.8	78.7	81.1	0.928

**Table 4:** The results of different methods.

Method	Acc%	Prec%	Rec%	F1%	AUC
SVM	61.3	39.4	56.4	46.4	0.639
MLP	75.5	58.9	57.9	58.4	0.792
ResNet-18 [36]	85.0	74.6	75.1	74.5	0.868
LSTM [37]	86.0	75.2	79.1	76.7	0.904
GCN [23]	84.3	72.3	74.1	73.2	0.862
ST-GCN [24]	86.6	79.3	74.4	76.3	0.889
2s-ST-GCN [17]	87.8	81.4	76.5	78.5	0.907
iTransformer [28]	85.5	73.1	**81.1**	76.8	0.908
JTFT [29]	88.1	80.4	79.6	79.8	0.916
**Ours**	**89.1**	**83.8**	78.7	**81.1**	**0.928**

**Table 5:** The results of ablation experiments.

Data Parallelism *	Cross-Attention	Mamba-2	Acc%	Prec%	Rec%	F1%	AUC
			87.4	81.1	75.2	78.0	0.903
✓			88.0	81.7	78.3	79.6	0.913
✓	✓		88.9	**85.0**	77.1	80.8	0.923
✓	✓	✓	**89.1**	83.8	**78.7**	**81.1**	**0.928**

* Data Parallelism indicates whether dual-channel processing is applied to data from bilateral camera sensors.

**Table 6:** The results of different $\lambda_{1}$ parameters.

Parameter	Acc%	Prec%	Rec%	F1%	AUC
$\lambda_{1}=0$	88.4	**84.3**	76.7	80.3	0.926
$\lambda_{1}=0.1$	87.9	80.3	77.2	77.9	0.909
$\lambda_{1}=0.01$	**89.1**	83.8	**78.7**	**81.1**	**0.928**

**Table 7:** The results of different $w_{1}$, $w_{2}$ parameters.

Parameter	Acc%	Prec%	Rec%	F1%	AUC
$w_{1}=1.0,w_{2}=1.0$	88.6	83.7	76.2	79.7	0.919
$w_{1}=0.6,w_{2}=1.4$	85.6	74.0	81.4	77.4	0.893
$w_{1}=0.9,w_{2}=1.1$	**89.1**	**83.8**	**78.7**	**81.1**	**0.928**

**Table 8:** Results of frontal- and lateral-view experiments.

View	Acc%	Prec%	Rec%	F1%	AUC
Only frontal view	85.5	74.1	78.7	76.2	0.905
Only lateral view	87.2	80.0	76.3	77.9	0.910
Frontal and lateral view	**89.1**	**83.8**	**78.7**	**81.1**	**0.928**

**Table 9:** Results of comparison with prior information.

Method	Acc%	Prec%	Rec%	F1%	AUC
Without prior information	89.1	83.8	**78.7**	81.1	**0.928**
With prior information	**89.7**	**86.3**	77.9	**81.8**	0.914

**Table 10:** Results of portable device in different usage modes.

Camera Sensor	Prior Information	Acc%	Prec%	Rec%	F1%	AUC
Only Lateral Camera		87.2	80.0	76.3	77.9	0.910
Only Lateral Camera	✓	87.7	80.1	77.2	78.6	0.898
Frontal and Lateral Camera		89.1	83.8	**78.71**	81.1	**0.928**
Frontal and Lateral Camera	✓	**89.7**	**86.3**	77.9	**81.8**	0.914

**Table 11:** Comparison with present studies.

Studies	Subjects (HC/PD)	View	Class	Method	Acc%	F1%	AUC
Guo et al. [17]	142 (-/142) *	Frontal	4	2s-ST-AGCN	65.7	64.9	0.860
Zeng et al. [18]	80 (26/54)	Lateral	3	ST-GCN + VGG	71.3	71.0	0.789
Endo et al. [20]	54 (-/-)	Lateral	4	GaitForeMer	-	76.0	-
Kaur et al. [21]	33 (14/19)	Frontal and Lateral	2	CNN	79.3	78.2	0.933
He et al. [19]	191 (95/96)	Frontal	2	ADGCN	84.1	85.8	-
Our Study	388 (304/84)	Frontal and Lateral	2	CMSA-Net	89.1	81.1	0.928

* If the content is “-”, it indicates that the original paper did not mention this portion of the data.

## Data Availability

The data presented in this study are available on request from the corresponding author. The data are not publicly available due to privacy and ethical restrictions.

## References

[1] Dorsey E.R., Sherer T., Okun M.S., Bloem B.R. (2018). The emerging evidence of the Parkinson pandemic. J. Park. Dis..

[2] Deliz J.R., Tanner C.M., Gonzalez-Latapi P. (2024). Epidemiology of Parkinson’s disease: An update. Curr. Neurol. Neurosci. Rep..

[3] Tolosa E., Garrido A., Scholz S.W., Poewe W. (2021). Challenges in the diagnosis of Parkinson’s disease. Lancet Neurol..

[4] Becker G., Müller A., Braune S., Büttner T., Benecke R., Greulich W., Klein W., Mark G., Rieke J., Thümler R. (2002). Early diagnosis of Parkinson’s disease. J. Neurol..

[5] Pagan F.L. (2012). Improving outcomes through early diagnosis of Parkinson’s disease. Am. J. Manag. Care.

[6] Bhidayasiri R., Martinez-Martin P. (2017). Clinical assessments in Parkinson’s disease: Scales and monitoring. Int. Rev. Neurobiol..

[7] Politis M. (2014). Neuroimaging in Parkinson disease: From research setting to clinical practice. Nat. Rev. Neurol..

[8] Rodriguez-Oroz M.C., Jahanshahi M., Krack P., Litvan I., Macias R., Bezard E., Obeso J.A. (2009). Initial clinical manifestations of Parkinson’s disease: Features and pathophysiological mechanisms. Lancet Neurol..

[9] Mirelman A., Bonato P., Camicioli R., Ellis T.D., Giladi N., Hamilton J.L., Hass C.J., Hausdorff J.M., Pelosin E., Almeida Q.J. (2019). Gait impairments in Parkinson’s disease. Lancet Neurol..

[10] Wu P., Cao B., Liang Z., Wu M. (2023). The advantages of artificial intelligence-based gait assessment in detecting, predicting, and managing Parkinson’s disease. Front. Aging Neurosci..

[11] Di Biase L., Summa S., Tosi J., Taffoni F., Marano M., Rizzo A.C., Vecchio F., Formica D., Di Lazzaro V., Di Pino G. (2018). Quantitative analysis of bradykinesia and rigidity in Parkinson’s disease. Front. Neurol..

[12] Shi L.F., Yan X., Zhou W., Shi Y. (2024). Simple and efficient step detection algorithm for foot-mounted IMU. Meas. Sci. Technol..

[13] Lancini M., Serpelloni M., Pasinetti S., Guanziroli E. (2016). Healthcare sensor system exploiting instrumented crutches for force measurement during assisted gait of exoskeleton users. IEEE Sens. J..

[14] Zhou J., Mao Q., Yang F., Zhang J., Shi M., Hu Z. (2024). Development and assessment of artificial intelligence-empowered gait monitoring system using single inertial sensor. Sensors.

[15] Shi L.F., Liu Z.Y., Zhou K.J., Shi Y., Jing X. (2023). Novel deep learning network for gait recognition using multimodal inertial sensors. Sensors.

[16] Alazeb A., Batool M., Al Mudawi N., Alshehri M.S., Almakdi S., Almujally N.A., Algarni A. (2024). Effective gait abnormality detection in Parkinson’s patients for multi-sensors surveillance system. IEEE Access.

[17] Guo R., Shao X., Zhang C., Qian X. (2021). Multi-scale sparse graph convolutional network for the assessment of Parkinsonian gait. IEEE Trans. Multimed..

[18] Zeng Q., Liu P., Yu N., Wu J., Huo W., Han J. (2023). Video-based quantification of gait impairments in Parkinson’s disease using skeleton-silhouette fusion convolution network. IEEE Trans. Neural Syst. Rehabil. Eng..

[19] He Y., Yang T., Yang C., Zhou H. (2022). Integrated equipment for Parkinson’s disease early detection using graph convolution network. Electronics.

[20] Endo M., Poston K.L., Sullivan E.V., Fei-Fei L., Pohl K.M., Adeli E. Gaitforemer: Self-supervised pre-training of transformers via human motion forecasting for few-shot gait impairment severity estimation. Proceedings of the International Conference on Medical Image Computing and Computer-Assisted Intervention.

[21] Kaur R., Motl R.W., Sowers R., Hernandez M.E. (2022). A vision-based framework for predicting multiple sclerosis and Parkinson’s disease gait dysfunctions—A deep learning approach. IEEE J. Biomed. Health Inform..

[22] Hoehn M.M., Yahr M.D. (1967). Parkinsonism: Onset, progression, and mortality. Neurology.

[23] Kipf T.N., Welling M. (2016). Semi-supervised classification with graph convolutional networks. arXiv.

[24] Yan S., Xiong Y., Lin D. Spatial temporal graph convolutional networks for skeleton-based action recognition. Proceedings of the AAAI Conference on Artificial Intelligence.

[25] Ciresan D.C., Meier U., Masci J., Gambardella L.M., Schmidhuber J. Flexible, high performance convolutional neural networks for image classification. Proceedings of the Twenty-Second International Joint Conference on Artificial Intelligence.

[26] Simonyan K., Zisserman A. (2014). Very deep convolutional networks for large-scale image recognition. arXiv.

[27] Vaswani A., Shazeer N., Parmar N., Uszkoreit J., Jones L., Gomez A.N., Kaiser Ł., Polosukhin I. Attention is all you need. Proceedings of the Advances in Neural Information Processing Systems NIPS 2017.

[28] Liu Y., Hu T., Zhang H., Wu H., Wang S., Ma L., Long M. iTransformer: Inverted Transformers Are Effective for Time Series Forecasting. Proceedings of the Twelfth International Conference on Learning Representations.

[29] Chen Y., Liu S., Yang J., Jing H., Zhao W., Yang G. (2024). A joint time-frequency domain transformer for multivariate time series forecasting. Neural Netw..

[30] Dao T., Gu A. Transformers are SSMs: Generalized models and efficient algorithms through structured state space duality. Proceedings of the 41st International Conference on Machine Learning.

[31] Wang C.Y., Bochkovskiy A., Liao H.Y.M. YOLOv7: Trainable bag-of-freebies sets new state-of-the-art for real-time object detectors. Proceedings of the IEEE/CVF Conference on Computer Vision and Pattern Recognition.

[32] Krishnan S.R., Seelamantula C.S. (2012). On the selection of optimum Savitzky-Golay filters. IEEE Trans. Signal Process..

[33] Gretton A., Borgwardt K.M., Rasch M.J., Schölkopf B., Smola A. (2012). A kernel two-sample test. J. Mach. Learn. Res..

[34] Loshchilov I., Hutter F. Decoupled Weight Decay Regularization. Proceedings of the International Conference on Learning Representations.

[35] Kalra D.S., Barkeshli M. (2024). Why warmup the learning rate? Underlying mechanisms and improvements. Adv. Neural Inf. Process. Syst..

[36] He K., Zhang X., Ren S., Sun J. Deep residual learning for image recognition. Proceedings of the IEEE Conference on Computer Vision and Pattern Recognition.

[37] Guo Q., He Z., Wang Z. (2024). Monthly climate prediction using deep convolutional neural network and long short-term memory. Sci. Rep..

[38] Zhu J., Cui Y., Zhang J., Yan R., Su D., Zhao D., Wang A., Feng T. (2024). Temporal trends in the prevalence of Parkinson’s disease from 1980 to 2023: A systematic review and meta-analysis. Lancet Healthy Longev..

[39] Terrin F., Tesoriere A., Plotegher N., Dalla Valle L. (2023). Sex and brain: The role of sex chromosomes and hormones in brain development and Parkinson’s disease. Cells.

[40] Chen Z., Wu B., Li G., Zhou L., Zhang L., Liu J. (2023). Age and sex differentially shape brain networks in Parkinson’s disease. CNS Neurosci. Ther..

[41] Dumas R., Cheze L., Verriest J.P. (2007). Adjustments to McConville et al. and Young et al. body segment inertial parameters. J. Biomech..

[42] Ragonese P., D’Amelio M., Callari G., Aiello F., Morgante L., Savettieri G. (2007). Height as a potential indicator of early life events predicting Parkinson’s disease: A case-control study. Mov. Disord..

[43] Saari L., Backman E.A., Wahlsten P., Gardberg M., Kaasinen V. (2022). Height and nigral neuron density in Parkinson’s disease. BMC Neurol..

[44] Cersosimo M.G., Raina G.B., Pellene L.A., Micheli F.E., Calandra C.R., Maiola R. (2018). Weight loss in Parkinson’s disease: The relationship with motor symptoms and disease progression. BioMed Res. Int..

[45] Ma K., Xiong N., Shen Y., Han C., Liu L., Zhang G., Wang L., Guo S., Guo X., Xia Y. (2018). Weight loss and malnutrition in patients with Parkinson’s disease: Current knowledge and future prospects. Front. Aging Neurosci..

[46] Portugal B., Artaud F., Domenighetti C., Roze E., Degaey I., Canonico M., Elbaz A. (2023). Body mass index, abdominal adiposity, and incidence of Parkinson disease in French women from the E3N cohort study. Neurology.

[47] Kim H.J., Oh E.S., Lee J.H., Moon J.S., Oh J.E., Shin J.W., Lee K.J., Baek I.C., Jeong S.H., Song H.J. (2012). Relationship between changes of body mass index (BMI) and cognitive decline in Parkinson’s disease (PD). Arch. Gerontol. Geriatr..

[48] Palacios N., Gao X., McCullough M.L., Jacobs E.J., Patel A.V., Mayo T., Schwarzschild M.A., Ascherio A. (2011). Obesity, diabetes, and risk of Parkinson’s disease. Mov. Disord..

[49] Wang Y.L., Wang Y.T., Li J.F., Zhang Y.Z., Yin H.L., Han B. (2015). Body mass index and risk of Parkinson’s disease: A dose-response meta-analysis of prospective studies. PLoS ONE.

[50] Osler M., Okholm G.T., Villumsen M., Rozing M.P., Jørgensen T.S.H. (2022). Associations of young adult intelligence, education, height, and body mass index with subsequent risk of Parkinson’s disease and survival: A Danish cohort study. J. Park. Dis..

[51] Veer K., Pahuja S.K. (2022). Gender based assessment of gait rhythms during dual-task in Parkinson’s disease and its early detection. Biomed. Signal Process. Control.

